# The altering cellular components and function in tumor microenvironment during remissive and relapsed stages of anti-CD19 CAR T-cell treated lymphoma mice

**DOI:** 10.3389/fimmu.2023.1101769

**Published:** 2023-01-25

**Authors:** Kai Zhao, Chunxiao Ren, Donghai Tang, Li Zhao, Xianxian Chen, Ying Wang, Kailin Xu

**Affiliations:** ^1^ Blood Diseases Institute, Xuzhou Medical University, Xuzhou, Jiangsu, China; ^2^ Department of Hematology, The Affiliated Hospital of Xuzhou Medical University, Xuzhou, Jiangsu, China; ^3^ The Key Lab of Bone Marrow Transplantation, Xuzhou, Jiangsu, China

**Keywords:** chimeric antigen receptor T cell, CD19, tumor microenvironment, lymphoma, immunosuppression

## Abstract

Anti-CD19 chimeric antigen receptor (CAR) T cells represent a highly promising strategy for B-cell malignancies. Despite the inspiring initial achievement, remission in a notable fraction of subjects is short-lived, and relapse remains a major challenge. Tumor microenvironment (TME) was proved to be aroused by CAR T cells; however, little is known about the dynamic characteristics of cellular components in TME especially during the different phases of disease after anti-CD19 CAR T-cell treatment. We took advantage of an immunocompetent model receiving syngeneic A20 lymphoma cells to dissect the changes in TME with or without CAR T-cell injection. We found that anti-CD19 CAR T-cell treatment attenuated the symptoms of lymphoma and significantly prolonged mice survival through eradicating systemic CD19^+^ cells. Increased myeloid subsets, including CD11c^+^ DCs and F4/80^+^ macrophages with higher MHC II and CD80 expression in bone marrow, spleen, and liver, were detected when mice reached remission after anti-CD19 CAR T treatment. Compared to mice without anti-CD19 CAR T administration, intrinsic T cells were triggered to produce more IFN-γ and TNF-α. However, some lymphoma mice relapsed by day 42 after therapy, which coincided with CAR T-cell recession, decreased myeloid cell activation and increased Treg cells. Elevated intrinsic T cells with high PD-1 and TIGIT exhaust signatures and attenuated cytotoxicity in TME were associated with the late-stage relapse of CAR T-cell treatment. In summary, the cellular compositions of TME as allies of CAR T cells may contribute to the anti-tumor efficacy at the initial stage, whereas anti-CD19 CAR T-cell disappearance and host response immunosuppression may work together to cause lymphoma relapse after an initial, near-complete elimination phase.

## Introduction

Chimeric antigen receptor (CAR) T cellular therapy targeting the B-cell surface protein CD19 has produced remarkable outcomes in the treatment of B-cell malignancies (1–4). Approved anti-CD19 CAR therapies achieved objective response rates in over 80% of patients and complete responses in over 65% of patients who had exhausted all other treatment options, a unique achievement for CAR-T-cell therapies (5). In our clinical trials, anti-CD19 CAR T-cell therapies also demonstrated excellent activity in the treatment of leukemia, lymphoma, and multiple myeloma (6–8). Despite the inspiring initial achievement, remission in a notable fraction of subjects is short-lived and relapse remains a major challenge because 30%–60% of treated patients relapse within 1 year (9–11). Discovering the factors that affect tumor elimination after anti-CD19 CAR T-cell treatment is an essential step in developing strategies to prevent relapse.

Recently, evidence showed that two-thirds of tumor lysis events were directly performed by CAR T cells, which suggested CAR T-cell cytotoxicity was a major player in driving tumor regression but also suggested the existence of additional mechanisms elicited by CAR T cells in the tumor microenvironment (TME) (12). Although CAR T cells did not require antigen presentation and were directly activated without MHC restriction, the TME components, including antigen-presenting cells (APC) and endogenous T cells, might still be aroused after CAT T-cell treatment. After three days of anti-CD19 CAT T-cell injection, results demonstrated that the transfer of CAR T cells promoted the cytotoxic phenotype of host immune effectors and the upregulation of genes involved in antigen presentation in some myeloid subsets (13). Those data suggested that tumor progression was the result of a combination of CAR T cells and intrinsic immune responses induced by CAR T-cell infusion. So, in addition to the direct killing of CAR T cells, the role of the host immune system in response to therapy needs to be further investigated.

The paradoxical role of the TME during stages of tumor progression is dependent on its cellular components, which are highly heterogeneous and versatile (14). The generation of tumor-specific endogenous T cells and activated monocytes and macrophages was investigated in human IL13Rα2-CAR T-cell responding patients, suggesting the immune cells in TME may cooperate with CAR T therapy to improve therapeutic effectiveness (15). However, Treg cells are another TME cell type that play immunosuppressive roles on tumors, which suppress tumor-associated antigen presentation and also interfere with cytotoxic T-cell function by inhibiting cytolytic granule release (16). Tumor-associated macrophages (TAM) are important regulators of tumorigenesis, and they are functionally plastic, which can alter their polarization state to accommodate different physiological conditions (17). Therefore, we hypothesized that the cellular compositions and functions of TME might be diverse and dependent on different disease stages following anti-CD19 CAR T-cell treatment, which induces beneficial or adverse consequences for tumorigenesis.

To address this question, we took advantage of the immunocompetent mice bearing A20 lymphoma cells to dissect the changes of immune cells in TME, which probably will be remodified by anti-CD19 CAR T cells. We illustrated here that effective CD11b^+^ myeloid subsets and cytokine producing endogenous T cells accumulated in initial-remissive mice following anti-CD19 CAR T-cell administration. When the mice were resistant to CAR T cells, declining host immune responses characterized by reduced activity of macrophages and DCs as well as increased immunosuppressive Treg cells were investigated. Our results identify the potential mechanisms of lymphoma relapse as not only the disappearance of anti-CD19 CAR T cells but also intrinsic T-cell dysfunction in TME. Therefore, redressing the immunosuppressive TME represents an attractive strategy to prevent relapses after therapy.

## Materials and methods

### Mice

Female BALB/c mice, between 6–8 weeks of age and 22–25 g in weight, were obtained from the Beijing Vital River Laboratory Animal Technology. Mice were housed at a constant temperature of 25 ± 2 °C and a relative humidity of 55% in the animal house. These are specific pathogen free conditions at Xuzhou Medical University. Animal experiments were approved by the Medical Ethics Committee of Xuzhou Medical University, Xuzhou, Jiangsu, China. All mice were included in the experiment, and best efforts were made to minimize animal suffering. All the experimental procedures used in this study were carried out according to the experimental animal protocol approved by the ethics committee.

### Lymphoma mouse model establishment and evaluation

A20-GFP^+^ cell line was preserved in our laboratory, which was cultured in RPMI-1640 medium containing β-mercaptoethanol (37°C, 5% CO_2_ incubator), and the CD19 expression was detected by flow. BALB/c mice received a sublethal dose of irradiation (3.5 Gy) that was used as a conditioning regimen, and a B-cell lymphoma model was established by intravenously injecting A20-GFP^+^ cells (0.5 × 10^6^/mouse) 4 h later. The mice were monitored daily for survival and every 3 days for body weight changes. The clinical scores of the model were assessed based on three parameters, including weight loss, activity, and paralysis. Each parameter contains a severity scale, and the maximum score is 15, which is shown in (**Supplementary Table 1**).

### Anti-CD19 CAR plasmid extraction and retrovirus package

The plasmid of anti-CD19 CAR was kindly provided by Prof. Peng Li (Guangzhou Institute of Biomedicine and Health, Chinese Academy of Sciences). The CAR construct is composed of the anti-murine CD19 single-chain fragment variable domain, the transmembrane and intracellular domains of CD28, and the CD3z intracellular domain. The retroviral vector also encodes green fluorescent protein (GFP) to evaluate transduction efficiency and identify CAR T cells. DH5α competent cells were used in the plasmid amplification, and the extraction was done following the instructions of the plasmid extraction kit (Omega). The concentration and purity of the anti-CD19 CAR plasmid were tested, and the plasmid was stored at −20 °C. Plasmids (15 μg) and 45 μl of X-tremeGENE 9 DNA transfection reagent (Roche) were transfected into Plat-E cells to package retroviral anti-CD19 CAR. The fluorescence intensity of GFP was investigated with a fluorescence microscope (DP72, OLYMPUS). Retrovirus particles were filtered and stored at −80°C.

### CAR T-cell generation and administration

Splenic CD3^+^ T cells of BALB/c mice were isolated and purified using EasySep negative selection reagents according to the manufacturer’s instructions (StemCell Technologies). T-cell purity was >95%. Purified T cells (2 × 10^6^/ml) were activated in a 24-well plate pre-coated with anti-CD3 mAb (2 μg/ml, BioLegend) and anti-CD28 mAb (2 ug/ml, BioLegend) in the presence of hIL-2 (100 U/ml, Peprotech). Spin infections were performed 36 h after T-cell activation using retroviral particles (3 ml/well) supplemented with HitransG P virus infection enhancer (40 μl/ml, Genechem). T cells were cultured for 2 additional days in the presence of hIL-2. Infected T cells were collected and the percentage of CD3^+^GFP^+^ CAR T cells was detected by flow cytometry. Three days after the lymphoma model was established, mice were randomly divided into two groups that were either given a single dose of anti-CD19 CAR T cells (1 × 10^6^/mouse) through the tail vein or not. The CAR-T-cell treatment day was set as day 0.

### Cell extractions

Bone marrow (BM), spleen, and liver were harvested on days 9, 15, and 42. BM cells were isolated by flushing the femurs and tibias of tumor-bearing mice. The spleen was ground, and single-cell suspensions were prepared by filtering the cells through 200 um cell strainers. Livers were excised, minced, and passed through a cell strainer. Suspensions were centrifuged at 30*g* for 4 min at 4 °C to pellet nonhematopoietic cells. The supernatant was transferred to new tubes and spun at 460 g for 10 min. The cell pellet was resuspended in RPMI-1640, and lymphocytes were separated on a 25% Optiprep gradient (StemCell).

### Flow cytometry and antibodies

Single-cell suspensions from BM, spleen, and liver were stained with the following mAbs purchased from Biolegend: CD3 PE (Cat#100308, RRID: AB_312673), CD4 PE/Cyanine7 (Cat#100422, RRID: AB_312707), CD4 BV421 (Cat#100544, RRID: AB_11219790), CD8 APC/Cyanine7 (Cat#100714, RRID: AB_312753), CD11b PB (Cat#101224, RRID: AB_755986), F4/80 BV605 (Cat#123133, RRID: AB_2562305), MHCII AF700 (Cat#107622, RRID: AB_493727), CD19 PE-cf594 (Cat#115554, RRID: AB_2564001), FOXP3 AF647 (Cat#320014, RRID: AB_439750), CD11c PE-cf594 (Cat#117347, RRID: AB_2563654), IFN-γ PE/Cyanine7 (Cat#505826, RRID: AB_2295770), TNF-α APC (Cat#554420, RRID: AB_398553), CD25 PE (Cat#101904, RRID: AB_312847), PD-1 PE/Cyanine7 (Cat# 109109, RRID : AB_572016), TIGIT PE (Cat# 142104, RRID: AB_10933258), and Tim-3 BV605 (Cat# 119721, RRID: AB_2616907). CD3 EV450 (Elabscience, Cat#E-AB-F1013UQ) and CD80 PE (BD Bioscience, Cat#12-0801-81, RRID: AB_465751) were purchased from Elabscience and BD Bioscience, respectively. Granzyme B eFlourTM450 (Cat# 48-8898-82, RRID: AB_11149362) was purchased from Thermo Fisher Scientific. For intracellular cytokine staining, cells were stimulated with PMA (50 ng/ml, Sigma) and Ionomycine (750 ng/ml, Sigma) in the presence of Brefeldin A (10 μg/ml, Invitrogen) at 37 °C for 4 h. Flow cytometry was performed using a BD LSRFortessa Fortessa Flow Cytometer (RRID : SCR_019601). Data were analyzed using FlowJo software (FlowJo X, BD Inc. RRID : SCR_008520).

### *In vitro* cell co-culture

Splenocytes (1 × 10^6^/ml) from BALB/c mice were stained with Cell Trace Far Red Cell Proliferation Kit reagent (1 mM/ml, Invitrogen) and incubated in a 37°C water bath for 20 min. Then cells were washed in RPMI 1640 complete medium and incubated in a 37°C water bath in the dark for 5 min. After centrifugation at 400*g* for 5 min, dye labeled splenocytes were re-suspended to the working concentration (5 × 10^5^/ml), and co-cultured with A20 (5 × 10^5^/ml) and CAR-T cells (1 × 10^6^/ml) at the ratio of 1:1:2. Experimental groups were set as CAR-T + A20 + SP, A20 + SP, and SP alone. After 48 h of incubation, the cells were collected for flow cytometry detection. The dye stained spenocytes (APC^+^) were distinguished from *in vitro*-induced CAR-T cells (APC^−^GFP^+^CD3^+^) and activated T cells (APC^−^GFP^−^CD3^+^).

### Statistics

Dot plots were generated using FlowJo Software v10.6.2. Statistical analyses and graphs were done using GraphPad Prism 9 software (GraphPad Prism, San Diego, RRID : SCR_002798). Data conformed to a normal distribution and were presented as the mean ± standard deviation. The Student’s t test was used for comparison between two groups if the variance was homogeneous, and the non-parametric test was used for unequal variance. A one-way or two-way ANOVA was performed on multiple groups. Survival rates were compared using the log-rank test. A *P* value of <0.05 was considered statistically significant.

## Results

### Anti-CD19 CAR T-cell treatment attenuates the symptom of lymphoma mice

**Figure 1:**
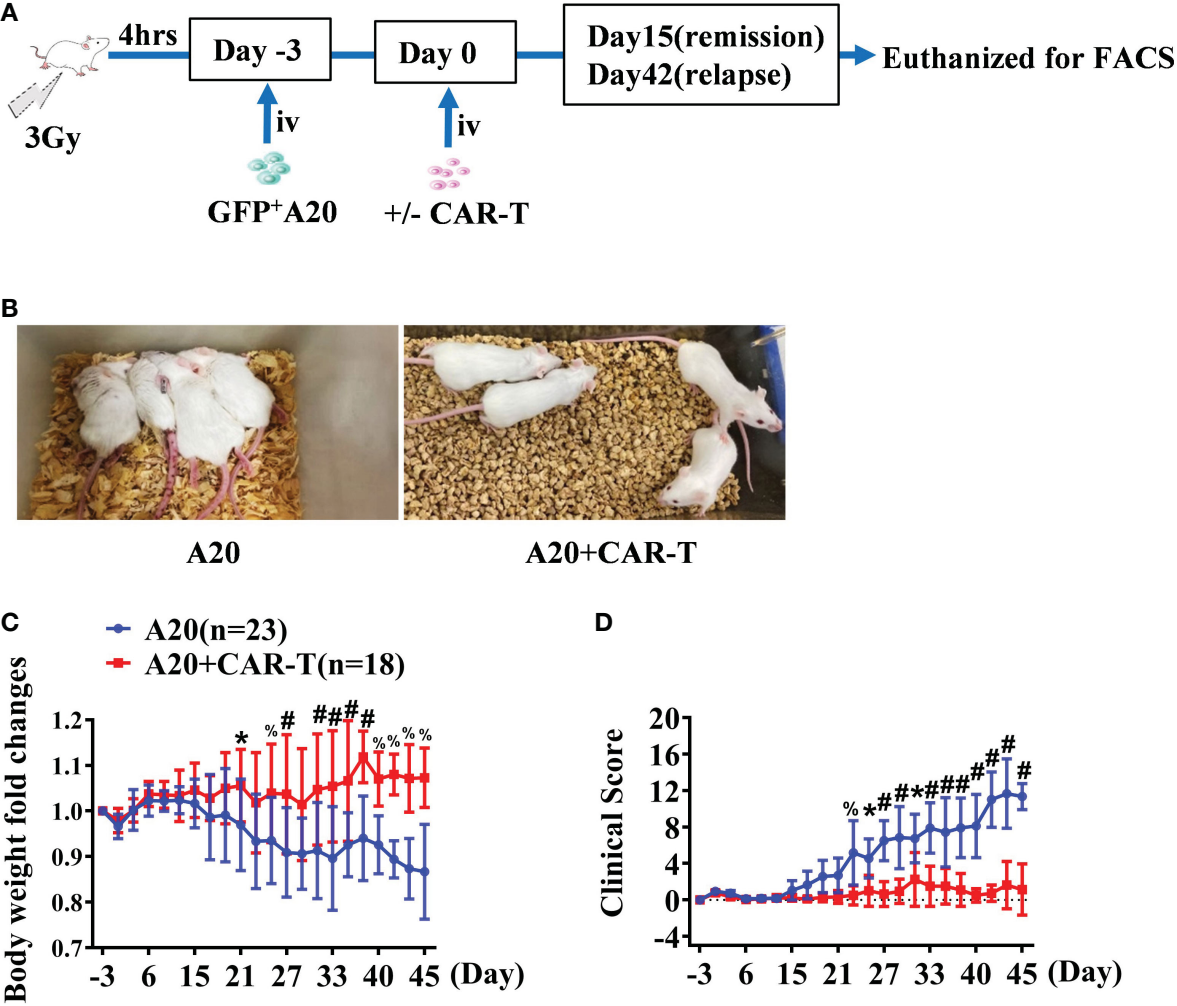
The general condition of anti-CD19 CAR T treated lymphoma mice. **(A)** The experiment design. **(B)** The state of the A20 induced lymphoma model. On day 32, the posture of mice injected with A20 cells combined with or without CD19 CAR-T cells were shown. **(C)** The body weight was tested every 2–3 days and the fold changes were analyzed. **(D)** Lymphoma models were evaluated, and the clinical score was recorded. Data were from two independent experiments. Two-way ANOVA was used to compare between the A20 (n = 23) and A20 + CAR-T groups (n = 18). *P < 0.01, ^%^ *P* < 0.001, ^#^ *P* < 0.0001.

With the aim to delineate the effect of anti-CD19 CAR T cell therapy, we used a syngeneic mouse model of A20 lymphoma as others and our previously described (18, 19). As shown in **Figure 1A**, when the mice treated with CAR T cells reached initial remission and then relapsed after CAR T-cell injection, the samples were harvested for detection on days 15 and 42, respectively. Firstly, mice were daily monitored for the occurrence of lymphoma symptoms, including weight loss, lethargy, and mobility. Compared to the asymptomatic mice after CAR T-cell treatment, reduced weights and mobility were investigated in the A20 mice since day 15. As shown in **Figure 1B**, significant lethargy and serious paralysis were present in A20 mice, while mice with CAR-T-cell treatment achieved remission. The body weights of mice treated with CAR-T cells gradually recovered from irradiation; however, the A20 group without CAR-T cells continued to reduce (**Figure 1C**). According to the standard of clinical scores, hosts with CAR-T cells displayed lower scores at the early time points, and the scores rose from day 31 because of the relapsed mice. However, obviously elevated clinical scores were shown in mice burdened with A20 cells alone since day 15, and the difference had statistical significance between groups with and without CAR T cells (**Figure 1D**).

### Anti-CD19 CAR T cells protect mice through eradicating systemic CD19^+^ tumor cells

**Figure 2:**
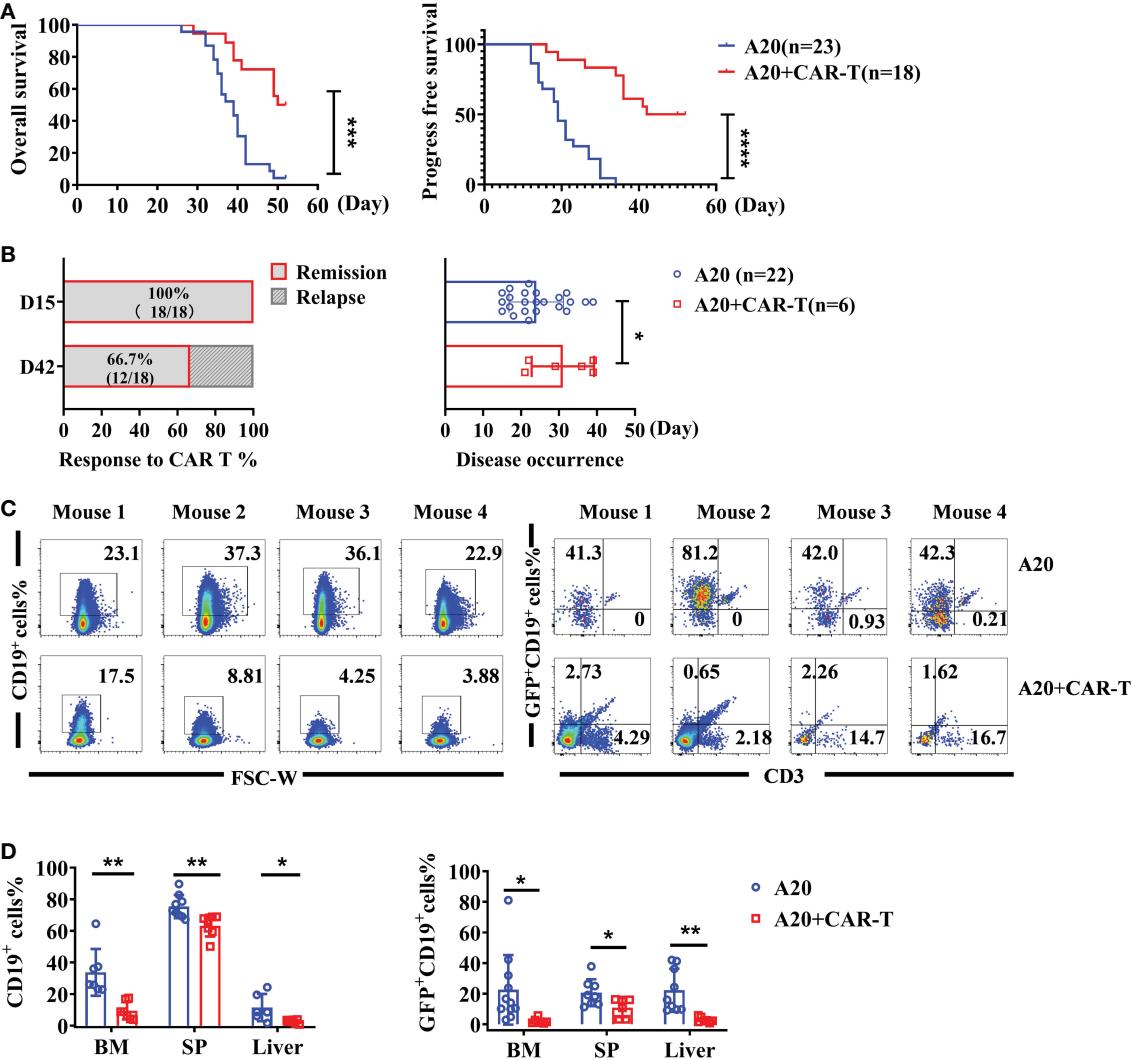
The effect of anti-CD19 CAR-T administration. **(A)** The survival of lymphoma mice. The OS and PFS data were recorded on the day of the CAR-T-cell injection. **(B)** The response rate after anti-CD19 CAR T-cell therapy and the time of disease occurrence were shown. **(C)** The percentage of total CD19^+^ cells and GFP^+^CD19^+^ cells (A20) of BM and liver on day 15. The representative flow cytometry plots were shown for four individual mice. **(D)** The statistics data for CD19^+^ cells and A20 cell percentage (n = 8 or 12). Survival was compared using the log-rank test, and the t-test was used to compare between two groups. There were two independent experiments that were repeated. *P < 0.05, **P < 0.01, ***P < 0.001, ****P < 0.0001.

Overall survival (OS) was significantly better in lymphoma hosts receiving anti-CD19 CAR T cells, and the benefits of CAR T-cell administration were enlarged in progress free survival (PFS) (**Figure 2A**). The response rate to anti-CD19 CAR T-cell therapy was calculated at the early and late stages. As shown in **Figure 2B**, the overall response rate was 100% (18/18), and all of them achieved remission on day 15. The cumulative incidence of relapse was 33.3% on day 42. Compared to A20 without CAR T cells, the onset of disease was delayed after therapy (**Figure 2B**). When CAR T-treated mice were in the remissive stage on day 15, as the target of CAR T cells, total CD19^+^ cells and GFP^+^ CD19^+^ lymphoma cells were detected. Representative flow data of BM in **Figure 2C** showed that the percentages of total CD19^+^ cells were reduced in mice with CAR T cells. Importantly, GFP^+^CD19^+^ malignant cells were dramatically eliminated by anti-CD19 CAR T cells, which was consistent with the remissive responses of CAR T-treated mice. Compared with A20 mice, the statistically analyzed data demonstrated that systemic CD19-expressing cells, and especially GFP^+^ CD19^+^ lymphoma cells, were significantly cleared after anti-CD19 CAR T-cell treatment (**Figure 2D**). These data confirmed the powerful protective effect of anti-CD19 CAR T cells on mice with lymphoma.

### Anti-CD19 CAR T cells promote myeloid cells recruitment and activation in mice achieved remission

**Figure 3:**
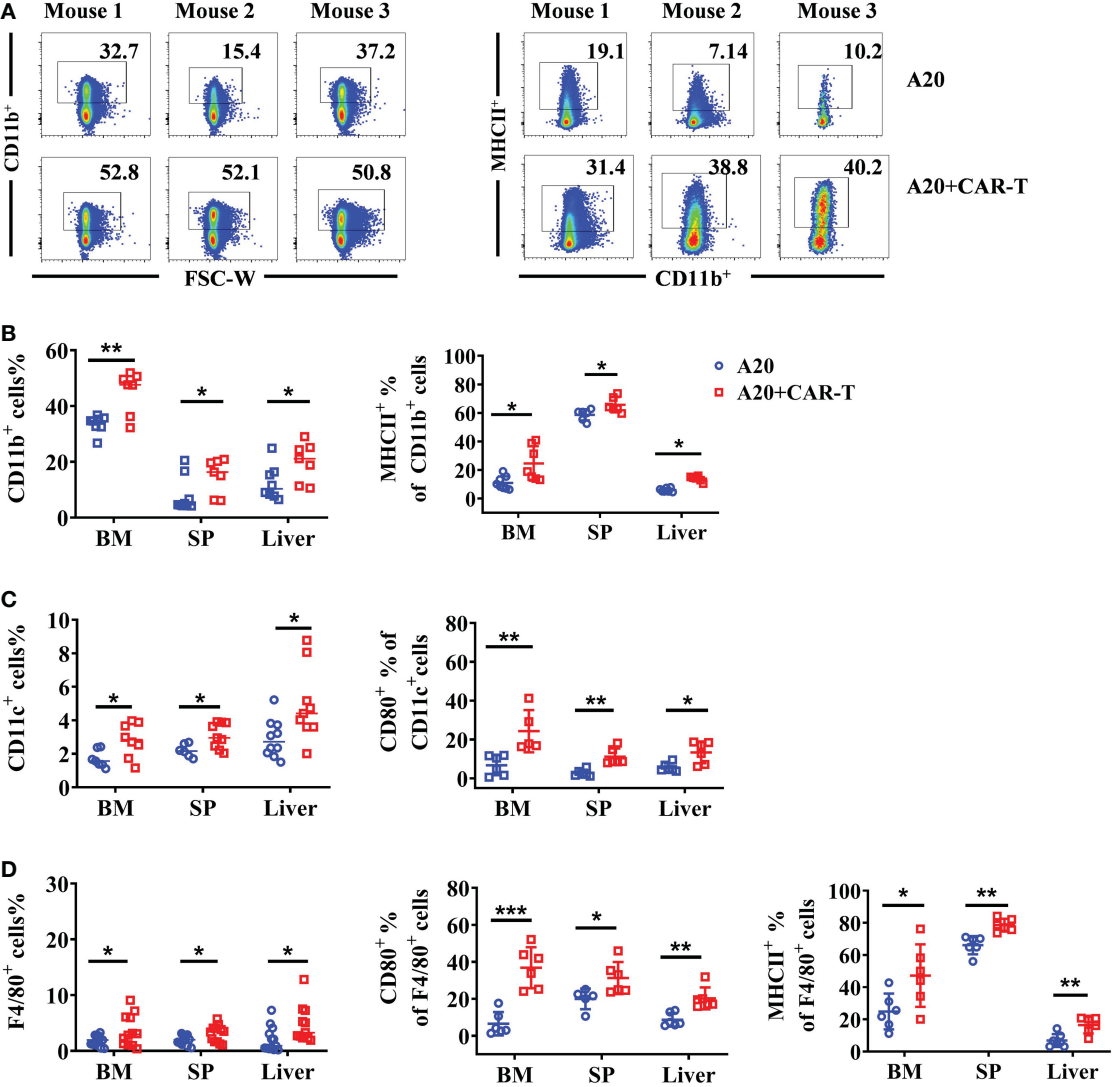
The myeloid cells were upregulated and activated in mice that achieved remission. On day 15, the BM, spleen, and liver from the lymphoma model with or without CAR-T cells were harvested. The myeloid cells were labeled with CD11b, CD11c, and F4/80. **(A)** The flow cytometry plot of CD11b+ mouse2 of A20 was corrected **(B–D)**. The percentage of CD11b^+^ myeloid cells, CD11c^+^ DCs, and F4/80^+^ macrophages. Then the expression of MHC II and CD80 in different populations were stained and analyzed by flow cytometry. Two independent experiments were repeated for **(B)** and **(C)**; Four independent experiments were done for **(D)**. Data were compared using unpaired Student’s t-test. *P < 0.05, **P < 0.01, ***P < 0.001.

Our results highlighted the importance of anti-CD19 CAR T cells in lymphoma cell clearance. At the remissive stage after anti-CD19 CAR T-cell treatment, the status of TME cells was to be explored in the next study. To do so, CD11b positive cells and their MHC II expression were detected on day 15 after CAR T cell treatment or untreatment. As shown in **Figure 3A**, more CD11b^+^ cells were found in CAR-T-cell administered mice, and the MHC II expression on CD11b^+^ cells in the BM were higher. Except BM, upregulated CD11b^+^ and higher MHC II expressed on CD11b^+^ cells were also detected in SP and liver, suggesting systemic activation of myeloid cells (**Figure 3B**). We also observed increased myeloid subsets, including CD11c^+^ DCs and F4/80^+^ macrophages in the BM, SP, and liver. Importantly compared to the A20 without CAR T-cell group, anti-CD19 CAR T cells triggered remarkable elevation of CD80 and MHC II levels in DCs and macrophages (**Figures 3C, D**). In summary, anti-CD19 CAR T-cell treatment modified the TME and induced a broad activation of the host myeloid immune cell system when the mice reached remission post-anti-CD19 CAR T-cell administration.

### Host T-cell functions are boosted following CAR T-cell treatment

**Figure 4:**
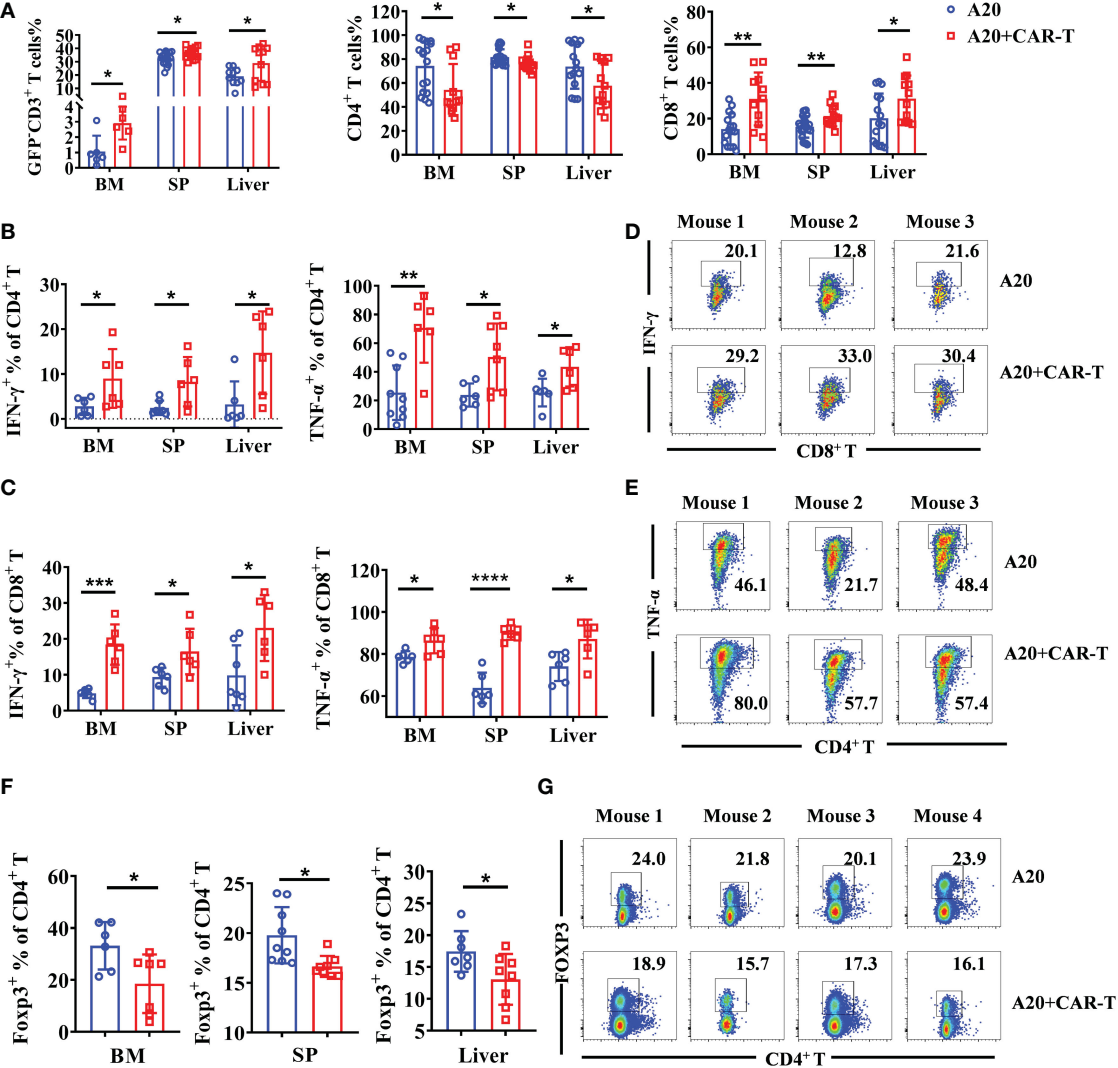
Intrinsic T cells were aroused in the remissive stage after anti-CD19 CAR T-cell treatment. **(A)** Percentage of CD3^+^ T cells and the subsets. **(B, C)** The cytokine expression on CD4^+^ and CD8^+^ T cells. **(D, E)** The representative flow data of IFN-γ and TNF-α produced CD8^+^ and CD4^+^ T cells from three mice. **(F)** The percentage of Treg cells in the BM, spleen, and liver were analyzed, and statistical analysis was done. **(G)** The representative data from SP on day 15 were shown. Cells were gated on CD4 and the expression of Foxp3 was shown. There were two or four repeated experiments. Data were mean ± SD and compared using unpaired Student’s *t-*test. *P < 0.05, **P < 0.01, ***P < 0.001, ****P < 0.0001.

When considering that all cells present in the TME might be involved in the CAR T-cell induced immune response, endogenous T cells were investigated when the lymphoma model reached remission. As shown in **Figure 4A**, the percentages of GFP^-^CD3^+^ T cells were higher in the anti-CD19 CAR T-cell treated group than in the untreated group. The increased cellular composition was primarily driven by CD8^+^ T cells in the BM, SP, and liver, whereas CD4^+^ T cells showed lower percentages (**Figure 4A**). Then, the functions of host T cells were evaluated by cytokine production. Compared to untreated mice, all the CD4^+^ and CD8^+^ T cells isolated from BM, SP, and liver produced much higher levels of IFN-γ and TNF-α in CAR T-cell treated mice, suggesting the enhanced cytotoxic characteristics of host T cells (**Figures 4B–E**). In addition, Treg cells labeled as Foxp3^+^ CD4^+^ T were widely decreased in CAR T-cell treated group than that in untreated group (**Figures 4F, G**). The data above supported the fact that anti-CD19 CAR T cells boosted the number and activity of cytotoxic T effectors, whereas they attenuated immunosuppressive Treg cells in the TME of initial remissive mice. The results suggested that at the early stage, when the lymphoma mice reached remission, endogenous effector T cells as allies of anti-CD19 CAR T cells possibly contributed to the anti-tumor efficacy.

### Syngeneic T cell are triggered in the process of CAR T-cell mediated tumor cytotoxicity

**Figure 5:**
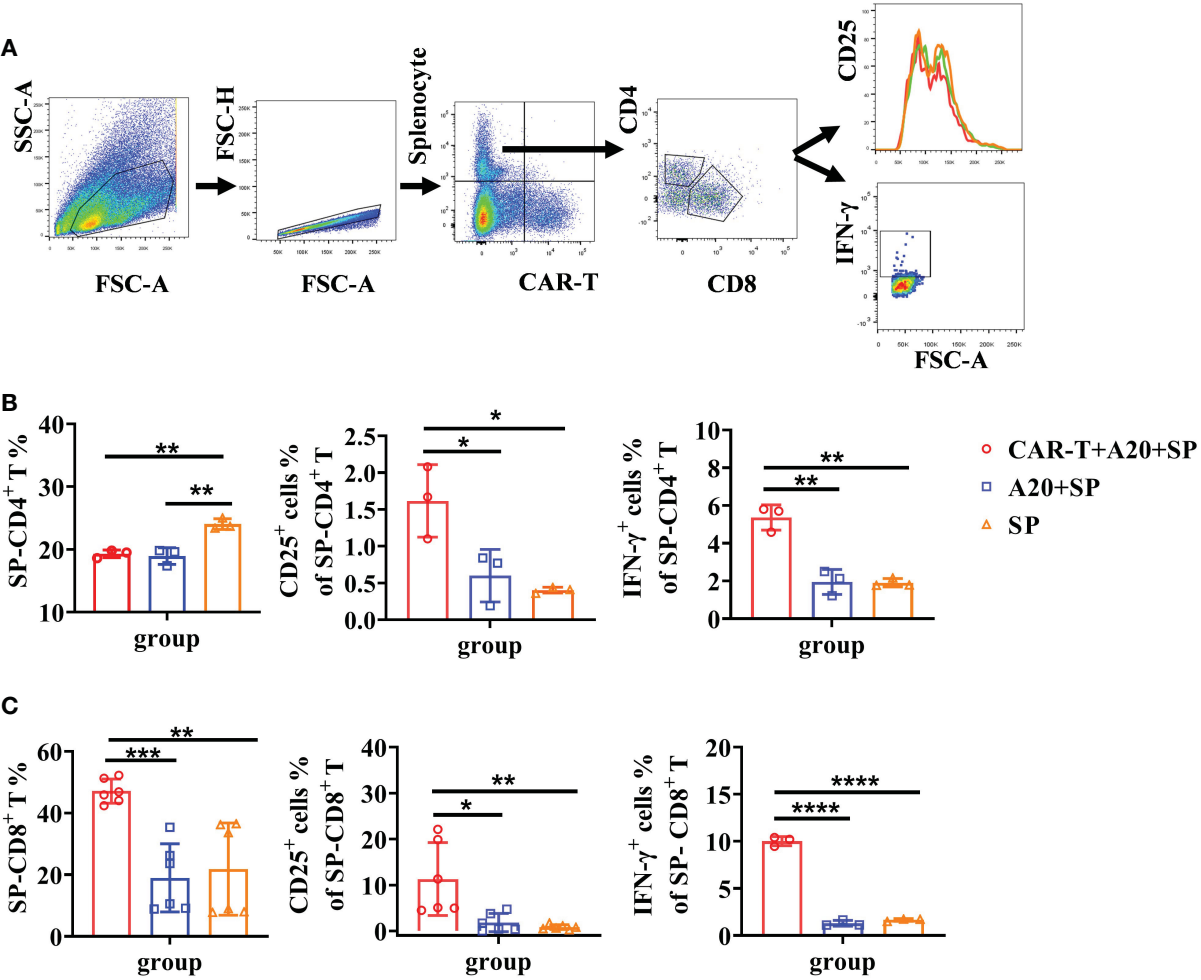
Elevated syngeneic T cell responses were induced by CD19 CAR-T triggered tumor lysis. Splenocytes from BALB/c mice were harvested and stained with Cell Trace Far Red. Syngeneic splenocytes, CD19 CAR-T, and A20 cells were co-cultured *in vitro*. Flow cytometry was done to detect T-cell activation and cytokine secretion. **(A)** Gating strategy of flow cytometry. **(B, C)** The percentage of T cells and the expression of IFN-γ. *P < 0.05, **P < 0.01, ***P < 0.001, ****P < 0.0001.

Based on the above findings, we next investigated whether syngeneic T-cell activation was implicated in the tumor lysis process by CAR T cells. To do so, BALB/c derived splenocytes (H-2^d^) were dyed with Celltrace Far Red fluorescein to distinguish them from anti-CD19 CAR T (H-2^d^) or A20 cells in the co-cultured system. The gating strategy for FACS data is shown in **Figure 5A**. Cells were harvested after 48 h incubation. Lower percentages of CD4^+^ T cells were found both in splenocytes plus A20 combined with or without CAR T cells than that in splenocytes alone group, which were consistent with the reduced CD4^+^ T cells *in vivo*. However, obvious increased CD25 expression and IFN-γ secretion of splenic CD4^+^ T cells were detected under the CAR T-cell induced A20 lysis condition (**Figure 5B**). As expected, CD8^+^ T cells of splenocytes showed a much higher percentage in the CAR T and A20 co-cultured system. In addition, significant upregulated CD25 levels and IFN-γ production were presented compared to the non-CAR T cell group (**Figure 5C**). Taken together, we confirmed that host T cells could be activated by CAR T-cell-induced anti-tumor efficacy *in vitro*. Furthermore, CD8^+^ T cells, which possess powerful tumor cytotoxicity, were more efficient at proliferation compared with CD4^+^ T cells.

### Tumor relapse coincides with CAR T-cell disappearance and immune inhibition

**Figure 6:**
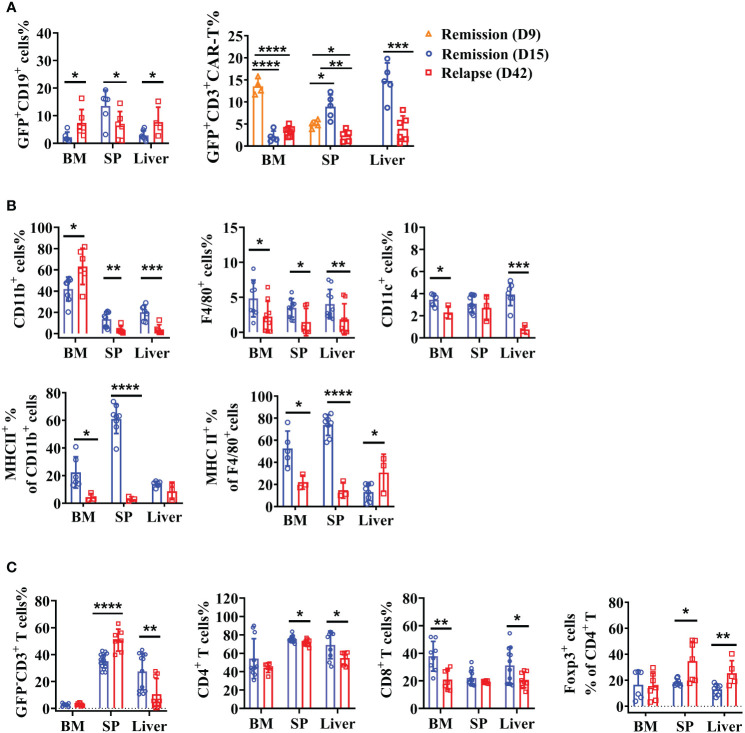
Recession of the intrinsic immune response with enhanced immunosuppression of TME in relapsed mice. After anti-CD19 CAR T-cell treatment for 15 and 42 days, lymphoma mice were in the remissive and relapsed stages, respectively. **(A)** The percentages of A20 and anti-CD19 CAR T cells were shown. **(B)** The quantity and expression of activation markers on myeloid cells were detected by flow cytometry. **(C)** The percentage of T-cell subsets. Two independent experiments were repeated. Data were mean ± SD and compared using unpaired Student’s t-test. *P < 0.05, **P < 0.01, ***P < 0.001, ****P < 0.0001.

Lymphoma mice treated with anti-CD19 CAR T cells exhibited significantly prolonged survival but some of them (33%) ultimately relapsed at the late time-points. By day 42 after therapy, the GFP^+^CD19^+^ tumor cells were obviously higher in the BM and liver of relapsed mice (**Figure 6A**), whereas no statistical difference was found in the GFP^-^CD19^+^ cells (data not shown). Furthermore, we confirmed the persistence of CAR T cells in the BM, spleen, and liver on days 9, 15, and 42 post-injection. However, CAR T cells became largely undetectable after treatment for 42 days (**Figure 6A**). To explore more cellular contexts of tumor relapsed TME, total CD11b^+^ myeloid cells, F4/80^+^ macrophages and CD11c^+^ DCs were detected. Compared to the percentages on day 15, the myeloid system cells, including macrophages and DCs, showed dramatically reduced numbers. Then the activated immune condition of myeloid cells and macrophages was assessed. As expected, significantly downregulated levels of MHC II on myeloid lineage cells and macrophages were detected in lymphoid organs (**Figure 6B**). Furthermore, we aimed to characterize the variation of host T-cell responses between the remissive and relapsed mice after CAR T therapy. Results in **Figure 6C** showed that lower percentages of CD4^+^ and CD8^+^ T cells were detected on day 42 than on day 15. Importantly, increased CD4^+^Foxp3^+^ Treg cells were observed in the spleen and liver compared the relapsed mice to the remissive ones (**Figure 6C**). Our results suggested that anti-CD19 CAR T-cell disappearance and immunosuppressive intrinsic immune cells of TME might contribute together to lymphoma relapse after an initial near-complete elimination phase.

### Relapse of anti-CD19 CAR T-cell therapy associates with TME exhausted T cells

**Figure 7:**
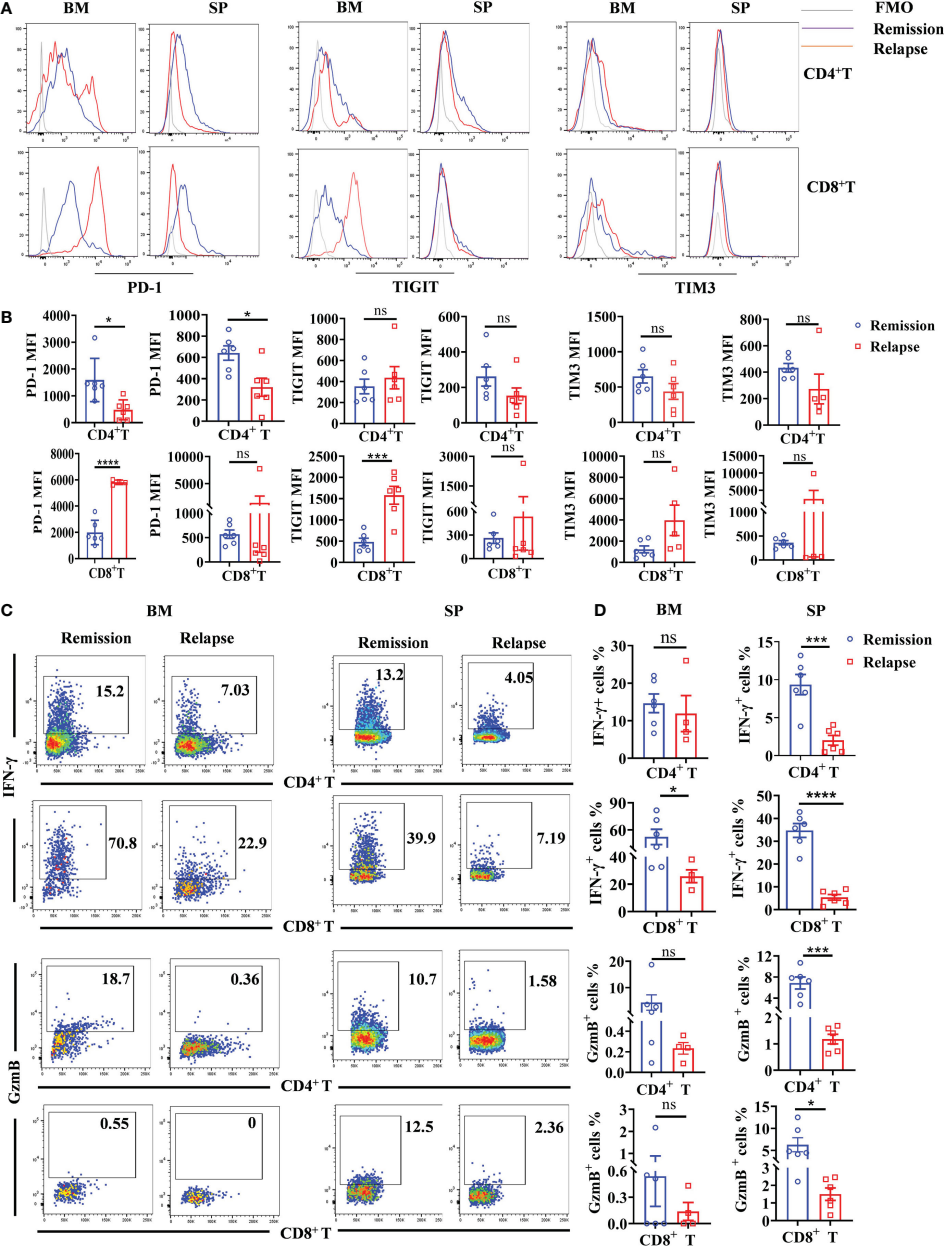
Increased exhausted characteristics and decreased cytotoxicity of intrinsic CD8^+^ T cells in the TME of relapsed mice. BM and spleen were harvested from lymphoma mice during the remissive and relapsed stages after anti-CD19 CAR T-cell treatment, respectively. **(A)** The representative fluorescence intensities of PD-1, TIGIT, and TIM3 on intrinsic T cells were shown. **(B)** The statistical differences between remissive and relapsed mice were compared. **(C, D)** The production of IFN-γ and GranzymeB by intrinsic T-cell subsets. Each symbol represents one individual mouse. Data were mean ± SD and compared using unpaired Student’s t-test. *P < 0.05, ***P < 0.001, ****P < 0.0001, ns *P >* 0.05.

T-cell exhaustion is characterized by high expression of inhibitory receptors and plays a dysfunctional role in tumor elimination (20). To further explore the TME associated causes of relapse after anti-CD19 CAR T-cell therapy, immune checkpoint molecules including PD-1, TIGIT, and TIM3 on intrinsic T cells were detected. As shown in **Figures 7A, B**, there was a small population of PD-1^high^ expressed CD4^+^ T cells in relapsed BM, whereas the total PD-1 fluorescence intensity was slightly decreased both in BM and spleen. By contrast, significantly increased PD-1 levels in CD8^+^ T cells were shown in relapsed BM. Consistent with the expression pattern of PD-1, TIGIT fluorescence intensity was obviously elevated in CD8^+^ T cells, and an increased trend was found in CD4^+^ T cells. However, there was no statistically significant difference in TIM3 between mice that achieved remission and relapse (**Figures 7A, B**). Next, we analyzed the cytotoxic functions of intrinsic T cells in remissive and relapsed TME. Comparing with T cells from remissive mice, CD8^+^ T cells from relapsed BM and spleen produced extremely lower levels of IFN-γ, whereas the similar expression pattern of IFN-γ in CD4^+^ T cells were observed (**Figures 7C, D**). GranzymeB, the cytotoxic molecular marker of effector T cells, was remarkably decreased both in CD4^+^ and CD8^+^ T cells in relapsed spleens, and reduced trends were found in BM (**Figures 7C, D**). The results may indicate that intrinsic T-cell exhaustion, especially in CD8^+^ T cells, and attenuated cytotoxicity are associated with a lack of a durable response to CAR T-cell therapy.

## Discussion

In this study, we demonstrated that disease progression in lymphoma mice was not only due to the direct cytotoxicity of anti-CD19 CAR T cells but also mingled with the contribution of TME. At the remission stage after CAR T-cell treatment, the triggered myeloid lineage cells and endogenous T cells effectively gave assistance to the CAR T cells to prolong their survival time. However, after the initial remission, ultimately relapse happened at the late phase of anti-CD19 CAR T administration. Mechanistically, it was potentially attributed to the exhausted CAR T cells, the weakened quantity and activity of myeloid cells and host T cells, combined with the increased immune inhibition of Treg cells.

Here, we proved the participation and diversity of TME during the antitumor process of anti-CD19 CAR T cells. However, the molecular mechanisms regulating anti-CD19 CAR T-cell activity and variant TME *in vivo* are still incompletely understood. Some data provided evidence that IFN-γ produced by CAR T cells was essential to boost the cytotoxic potential of CAR T cells and of host NK and T cells (13). Furthermore, IFN-γ responsiveness of host immune cells was critical for tumor immune landscape remodeling to promote a more activated and less suppressive TME (15). Except for the direct acting induced by IFN-γ receptor, long-range sensing of IFN-γ can modify tumor cells and the other cell types present in the TME (21). Our results showed that enhanced IFN-γ secretion was detected in anti-CD19 CAR T-cell treated remissive mice and in CAR T-cell stimulated syngeneic T cells *in vitro*. Therefore, it could be supposed that immune activity to attack tumors was primed by CAR T cells, and then endogenous T cells were triggered to strengthen the response. However, when the mice showed relapsed symptoms with the decline of anti-CD19 CAR T cells, dramatically decreased IFN-γ expression might contribute to the reduced host cytotoxic effectors and diminished tumor clearance. The fact is consistent with the results that IFN-γ regulates the TME by acting as a widespread and sustained cytokine field whose concentration is dependent on collective T cell activity (22). Thus, IFN-γ as an important mediator induces the interaction between anti-CD19 CAR T cells and the cellular compositions of TME. The diversity and immune characteristics of TME might be related to the IFN-γ level and the responsiveness of intrinsic immune cells.

In the TME, many cell types drive immunosuppression, including myeloid-derived suppressor cells, tumor-associated macrophages (TAMs), and regulatory T cells. TAMs are essential cellular components of TME and take part in diverse physiological and pathological processes (23, 24). TAMs are highly heterogeneous and plastic cell components of the TME, which can either promote tumor progression (M2-like) or boost anti-tumor immunity (M1-like) (25). Here, macrophages with higher CD80 and MHC II expression were found in the initial remission phase of CAR T treatment compared to the mice left untreated, whereas the mice relapsed with reduced MHC II levels. The data suggested that the variation and polarization switch of macrophages might occur during the anti-CD19 CAR T-cell mediated tumor cytotoxicity. The stimulation provided by the TME and the tumor itself is mostly responsible for the transformation of multiple TAM phenotypes (26). Michael et al. showed that macrophages in the TME of large B-cell lymphoma had the strongest association with non-durable responders after CD19 CAR T-cell treatment (27). However, the conflicting roles of macrophages mediated the synergistic or antagonistic effect on CAR T cell killing are probably due to spatiotemporal disparities and remain to be precisely determined in the future. Treg cells are involved in tumor development and progression by inhibiting antitumor immunity. Recently, data showed that Treg cells interacted with dendritic cells and modulated their numbers through a CTLA-4 and CD28-dependent feedback loop in the TME (28). Our results illustrate here that Treg cell numbers increased when mice relapsed after anti-CD19 CAR T-cell treatment. The upregulated Treg cells may inhibit the function of CAR T cells and intrinsic effector T cells, leading to the proliferation of lymphoma cells, and then a TME filled with malignancy cells is more conducive to the generation of Treg. It is well known that a high infiltration by Treg cells is associated with poor survival in various types of cancer (29). Thus, our data indicated that the increased number of Treg at the late stage was another potential mechanism of anti-CD19 CAR T-cell therapy failure.

Abundant reports showed that the memory-like or exhausted characteristics of CAR T cells were related to therapeutic success or failure, respectively (30, 31). Short-term CAR T-cell persistence is one of the main causes of disease relapse. It has been demonstrated that pre-conditioning modified the immunosuppressive TME and resulted in durable curative responses to PSCA-CAR T cells (32). On the other hand, it has been hypothesized that the development of T-cell senescence and CAR T exhaustion is triggered by co-inhibitory pathways in the TME (33). Consistent with the previous study, we found that the gradual disappearance of anti-CD19 CAR T cells were accompanied by increased Treg cells and decreased myeloid cell activation, which suggested a strong relationship between CAR T fate and the TME. Except for the observations directly on the CAR T cell, the roles of extrinsic factors in TME on the efficacy of CAR T treatment are in the early stages of exploration. The latest research demonstrated that higher post-treatment TME T-cell density reflected pre-treatment density and achieved complete remission in the ZUMA-1 trial. Furthermore, they revealed an association between poor CAR T-cell expansion and TME infiltration with exhausted Tc cells (34). In the current study, we indicated that intrinsic T cells are characterized by the expression of multiple exhausted markers and progressive loss of effector function. Thus, systemic T-cell exhaustion, especially CD8^+^ T cells, and injected CAR T cells are associated with the lack of a durable response that results in relapse.

In conclusion, we report the involvement of TME after anti-CD19 CAR T-cell treatment in lymphoma mice. The increased immunosuppressive functions of cellular components in TME are presented in the late phase of CAR T administration, which might promote disease relapse.

## Data availability statement

The original contributions presented in the study are included in the article/**Supplementary Material**. Further inquiries can be directed to the corresponding authors.

## Ethics statement

The animal study was reviewed and approved by Medical ethics committee of the Xuzhou Medical University, Xuzhou, Jiangsu, China.

## Author contributions

KX and KZ conceived of experiments, analyzed data, and revised the paper. KZ and CR conceived and executed experiments, analyzed data, and wrote the paper. KZ and CR contributed equally to this study. LZ, DT, and XC assisted in performing experiments. YW provided technical advice. All authors listed have made a substantial, direct, and intellectual contribution to the work and approved it for publication.

## References

[1] Porter DL Levine BL Kalos M Bagg A June CH. Chimeric antigen receptor-modified T cells in chronic lymphoid leukemia. New Engl J Med (2011) 365:725–33. doi: 10.1056/NEJMoa1103849 PMC3387277 21830940

[2] Maude SL Frey N Shaw PA Aplenc R Barrett DM Bunin NJ. Chimeric antigen receptor T cells for sustained remissions in leukemia. New Engl J Med (2014) 371:1507–17. doi: 10.1056/NEJMoa1407222 PMC4267531 25317870

[3] Porter DL Hwang WT Frey NV Lacey SF Shaw PA Loren AW. Chimeric antigen receptor T cells persist and induce sustained remissions in relapsed refractory chronic lymphocytic leukemia. Sci Trans Med (2015) 7:303ra139. doi: 10.1126/scitranslmed.aac5415 PMC5909068 26333935

[4] Zhang J Hu Y Yang J Li W Zhang M Wang Q. Non-viral, specifically targeted CAR-T cells achieve high safety and efficacy in b-NHL. Nature (2022) 609:369–74. doi: 10.1038/s41586-022-05140-y PMC9452296 36045296

[5] Voynova E Kovalovsky D. From hematopoietic stem cell transplantation to chimeric antigen receptor therapy: Advances, limitations and future perspectives. Cells (2021) 10:2845. doi: 10.3390/cells10112845 34831068 PMC8616322

[6] Cao J Wang G Cheng H Wei C Qi K Sang W. Potent anti-leukemia activities of humanized CD19-targeted chimeric antigen receptor T (CAR-T) cells in patients with relapsed/refractory acute lymphoblastic leukemia. Am J Hematol (2018) 93:851–8. doi: 10.1002/ajh.25108 29633386

[7] Sang W Shi M Yang J Cao J Xu L Yan D. Phase II trial of co-administration of CD19- and CD20-targeted chimeric antigen receptor T cells for relapsed and refractory diffuse large b cell lymphoma. Cancer Med (2020) 9:5827–38. doi: 10.1002/cam4.3259 PMC7433814 32608579

[8] Wang Y Cao J Gu W Shi M Lan J Yan Z. Long-term follow-up of combination of b-cell maturation antigen and CD19 chimeric antigen receptor T cells in multiple myeloma. J Clin oncol: Off J Am Soc Clin Oncol (2022) 40:2246–56. doi: 10.1200/JCO.21.01676 35333600

[9] Park JH Riviere I Gonen M Wang X Senechal B Curran KJ. Long-term follow-up of CD19 CAR therapy in acute lymphoblastic leukemia. New Engl J Med (2018) 378:449–59. doi: 10.1056/NEJMoa1709919 PMC6637939 29385376

[10] Bai Z Woodhouse S Zhao Z Arya R Govek K Kim D. Single-cell antigen-specific landscape of CAR T infusion product identifies determinants of CD19-positive relapse in patients with ALL. Sci Adv (2022) 8:eabj2820. doi: 10.1126/sciadv.abj2820 35675405 PMC9177075

[11] Westin JR Kersten MJ Salles G Abramson JS Schuster SJ Locke FL. Efficacy and safety of CD19-directed CAR-T cell therapies in patients with relapsed/refractory aggressive b-cell lymphomas: Observations from the JULIET, ZUMA-1, and TRANSCEND trials. Am J Hematol (2021) 96:1295–312. doi: 10.1002/ajh.26301 PMC9290945 34310745

[12] Cazaux M Grandjean CL Lemaitre F Garcia Z Beck RJ Milo I. Single-cell imaging of CAR T cell activity. Vivo reveals extensive Funct anat heterogen J Exp Med (2019) 216:1038–49. doi: 10.1084/jem.20182375 PMC6504219 30936262

[13] Boulch M Cazaux M Loe-Mie Y Thibaut R Corre B Lemaitre F. A cross-talk between CAR T cell subsets and the tumor microenvironment is essential for sustained cytotoxic activity. Sci Immunol (2021) 6:eabd4344. doi: 10.1126/sciimmunol.abd4344 33771887

[14] Quail DF Joyce JA. Microenvironmental regulation of tumor progression and metastasis. Nat Med (2013) 19:1423–37. doi: 10.1038/nm.3394 PMC3954707 24202395

[15] Alizadeh D Wong RA Gholamin S Maker M Aftabizadeh M Yang X. IFNgamma is critical for CAR T cell-mediated myeloid activation and induction of endogenous immunity. Cancer Discovery (2021) 11:2248–65. doi: 10.1158/2159-8290.CD-20-1661 PMC8561746 33837065

[16] von Boehmer H Daniel C. Therapeutic opportunities for manipulating T(Reg) cells in autoimmunity and cancer. Nat Rev Drug Discovery (2013) 12:51–63. doi: 10.1038/nrd3683 23274471

[17] DeNardo DG Ruffell B. Macrophages as regulators of tumour immunity and immunotherapy. Nat Rev Immunol (2019) 19:369–82. doi: 10.1038/s41577-019-0127-6 PMC7339861 30718830

[18] Kueberuwa G Zheng W Kalaitsidou M Gilham DE. & Hawkins, R.E. a syngeneic mouse b-cell lymphoma model for pre-clinical evaluation of CD19 CAR T cells. J Vis Exp (2018) 16:58492. doi: 10.3791/58492 PMC6235544 30394400

[19] Pan B Shang L Liu C Gao J Zhang F Xu M. PD-1 antibody and ruxolitinib enhances graft-versus-lymphoma effect without increasing acute graft-versus-host disease in mice. Am J Transplant (2021) 21:503–14. doi: 10.1111/ajt.16275 32805756

[20] Wherry EJ Kurachi M. Molecular and cellular insights into T cell exhaustion. Nat Rev Immunol (2015) 15:486–99. doi: 10.1038/nri3862 PMC4889009 26205583

[21] Hoekstra ME Bornes L Dijkgraaf FE Philips D Pardieck IN Toebes M. Long-distance modulation of bystander tumor cells by CD8(+) T cell-secreted IFNgamma. Nat Cancer (2020) 1:291–301. doi: 10.1038/s43018-020-0036-4 32566933 PMC7305033

[22] Thibaut R Bost P Milo I Cazaux M Lemaitre F Garcia Z. Bystander IFN-gamma activity promotes widespread and sustained cytokine signaling altering the tumor microenvironment. Nat Cancer (2020) 1:302–14. doi: 10.1038/s43018-020-0038-2 PMC7115926 32803171

[23] Mantovani A Marchesi F Malesci A Laghi L Allavena P. Tumour-associated macrophages as treatment targets in oncology. Nat Rev Clin Oncol (2017) 14:399–416. doi: 10.1038/nrclinonc.2016.217 28117416 PMC5480600

[24] Mantovani A Allavena P Marchesi F Garlanda C. Macrophages as tools and targets in cancer therapy. Nat Rev Drug Discovery (2022) 21:799–820. doi: 10.1038/s41573-022-00520-5 PMC9380983 35974096

[25] Rodriguez-Garcia A Lynn RC Poussin M Eiva MA Shaw LC O'Connor RS. CAR-T cell-mediated depletion of immunosuppressive tumor-associated macrophages promotes endogenous antitumor immunity and augments adoptive immunotherapy. Nat Commun (2021) 12:877. doi: 10.1038/s41467-021-20893-2 33563975 PMC7873057

[26] Zhang J Zhou X Hao H. Macrophage phenotype-switching in cancer. Eur J Pharmacol (2022) 931:175229. doi: 10.1016/j.ejphar.2022.175229 36002039

[27] Jain MD Zhao H Wang X Atkins R Menges M Reid K. Tumor interferon signaling and suppressive myeloid cells are associated with CAR T-cell failure in large b-cell lymphoma. Blood (2021) 137:2621–33. doi: 10.1182/blood.2020007445 PMC8120145 33512407

[28] Marangoni F Zhakyp A Corsini M Geels SN Carrizosa E Thelen M. Expansion of tumor-associated treg cells upon disruption of a CTLA-4-dependent feedback loop. Cell (2021) 184:3998–4015e3919. doi: 10.1016/j.cell.2021.05.027 34157302 PMC8664158

[29] Ohue Y Nishikawa H Regulatory T. (Treg) cells in cancer: Can treg cells be a new therapeutic target? Cancer Sci (2019) 110:2080–9. doi: 10.1111/cas.14069 PMC6609813 31102428

[30] Fraietta JA Lacey SF Orlando EJ Pruteanu-Malinici I Gohil M Lundh S. Determinants of response and resistance to CD19 chimeric antigen receptor (CAR) T cell therapy of chronic lymphocytic leukemia. Nat Med (2018) 24:563–71. doi: 10.1038/s41591-018-0010-1 PMC6117613 29713085

[31] Deng Q Han G Puebla-Osorio N Ma MCJ Strati P Chasen B. Characteristics of anti-CD19 CAR T cell infusion products associated with efficacy and toxicity in patients with large b cell lymphomas. Nat Med (2020) 26:1878–87. doi: 10.1038/s41591-020-1061-7 PMC8446909 33020644

[32] Murad JP Tilakawardane D Park AK Lopez LS Young CA Gibson J. Pre-conditioning modifies the TME to enhance solid tumor CAR T cell efficacy and endogenous protective immunity. Mol Ther (2021) 29:2335–49. doi: 10.1016/j.ymthe.2021.02.024 PMC8261088 33647456

[33] Kasakovski D Xu L Li Y. T Cell senescence and CAR-T cell exhaustion in hematological malignancies. J Hematol Oncol (2018) 11:91. doi: 10.1186/s13045-018-0629-x 29973238 PMC6032767

[34] Scholler N Perbost R Locke FL Jain MD Turcan S Danan C. Tumor immune contexture is a determinant of anti-CD19 CAR T cell efficacy in large b cell lymphoma. Nat Med (2022) 28:1872–82. doi: 10.1038/s41591-022-01916-x PMC9499856 36038629

