# Supplementary figures and images for: The altering cellular components and function in tumor microenvironment during remissive and relapsed stages of anti-CD19 CAR T-cell treated lymphoma mice
Source: Front Immunol. 2023 Jan 25;14:1101769. doi: 10.3389/fimmu.2023.1101769 (PMC9905118; doi:10.3389/fimmu.2023.1101769)

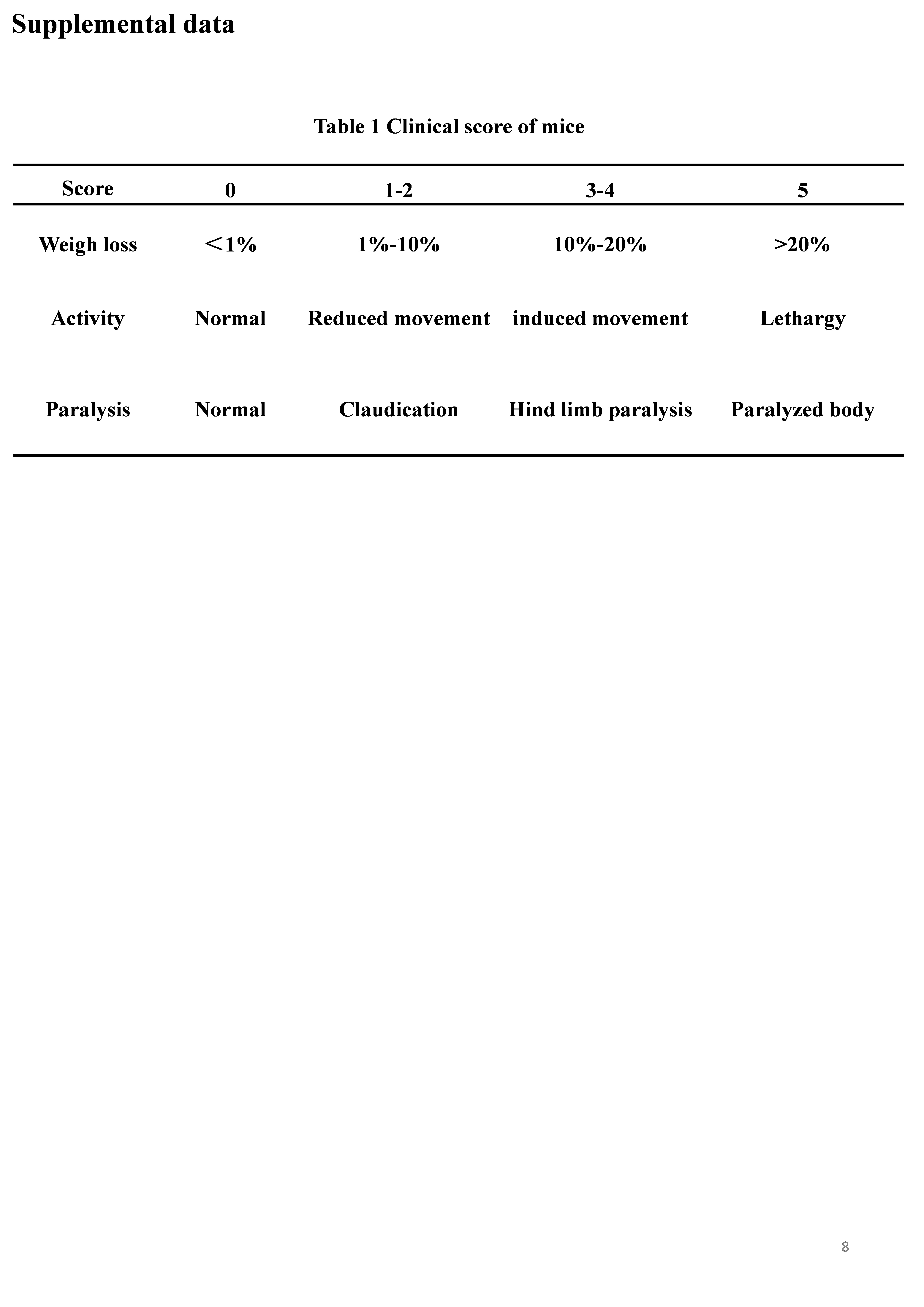

Supplement: Supplementary file 1 [file Image_1.jpeg]
